# Microwave- and Ultrasound-Assisted Extraction of Cucurbitane-Type Triterpenoids from *Momordica charantia* L. Cultivars and Their Antiproliferative Effect on SAS Human Oral Cancer Cells

**DOI:** 10.3390/foods11050729

**Published:** 2022-03-01

**Authors:** Yu-Tsung Lee, Li-Heng Pao, Chi-Yuan Chen, Sui-Qing Huang, Alaganandam Kumaran, Jong-Ho Chyuan, Chun-Hui Chiu

**Affiliations:** 1 Graduate Institute of Health Industry and Technology, Research Center for Chinese Herbal Medicine and Research Center for Food and Cosmetic Safety, College of Human Ecology, Chang Gung University of Science and Technology, No. 261, Wenhua 1st Road, Guishan District, Taoyuan City 33303, Taiwan; ytlee@mail.cgust.edu.tw (Y.-T.L.); paolhaa@gmail.com (L.-H.P.); cychen@mail.cgust.edu.tw (C.-Y.C.); sqhuang@mail.cgust.edu.tw (S.-Q.H.); 2 Department of Gastroenterology and Hepatology, Chang Gung Memorial Hospital, Guishan District, Taoyuan City 33303, Taiwan; 3 Tissue Bank, Chang Gung Memorial Hospital, Guishan District, Taoyuan City 33303, Taiwan; 4 Chemical Sciences and Technology Division, CSIR-National Institute for Interdisciplinary Science and Technology (NIIST), Thiruvananthapuram, Kerala City 695019, India; akumaran@niist.res.in; 5 Hualien District Agricultural Research and Extension Station, Hualien City 973044, Taiwan; jonghoc@hdares.gov.tw; 6 Department of Traditional Chinese Medicine, Chang Gung Memorial Hospital, Keelung City 20401, Taiwan

**Keywords:** *Momordica charantia* L., microwave-assisted extraction, 3β,7β,25-trihydroxycucurbita-5,23(E)-dien-19-al, momordicine I, UHPLC–MS/MS, SAS cell lines

## Abstract

Cucurbitane-type triterpenoids are a major class of bioactive compounds present in bitter melon. In the present study, six different cultivars of bitter melon were extracted by using microwave- or ultrasound-assisted techniques to identify the prominent method that can extract the majority of cucurbitane-type triterpenoids. A UHPLC–MS/MS (ultra-high-performance liquid chromatography tandem mass spectrometry) system was used for the identification and quantification of ten cucurbitane-type triterpenoids. The results suggest that the use of microwave-assisted extraction on cultivars 4 and 5 produced higher amounts of the selected cucurbitane-type triterpenoids. The interpretation of principal component analysis also identified that cultivar 4 is significantly different from the others in which the compounds 3β,7β,25-trihydroxycucurbita-5,23(E)-dien-19-al and momordicine I were found in higher quantities. Upon further evaluation, it was also identified that these two triterpenoids can act as antiproliferative agents due to their effects on SAS human oral cancer cell lines.

## 1. Introduction

Even with the significant improvements and advancements in therapies in recent years, cancer remains the second leading cause of death. According to the latest global statistics, it is estimated that more than 22 million new cancer cases will develop by 2030. Oral squamous cell carcinoma (OSCC) is one of the common cancers in Taiwan and worldwide [1,2]. The death rate associated with oral cancer is significantly higher than that of other cancers, not due to the fact that it is difficult to diagnose or discover but due to the cancer being routinely discovered late in its development [2,3]. Even though potential target-based therapies are available in proper dosing or as combination treatment protocols, these methods still exert side effects and are expensive. Recent reports from the World Health Organization suggest that around 80% of the world population are dependent on using traditional herbal medicine for their primary health care needs. Several fruits and vegetables have also been reported to play major roles in reducing cancer risk, where the cumulative effects of their bioactive metabolites along with several vitamins, minerals, proteins, and fibers are biologically essential. Hence, numerous studies have been devoted to finding novel agents, especially from natural plants that can reduce the risk of oral cancer development or trigger the apoptosis of tumor cells [4]. In addition, it is well known that the 5β,19-epoxy ring and C (25)-*O*-methyl in cucurbitane-type triterpenoid structures show prominent antiproliferative effects [5]. For example, cucurbitacin E from the climbing stem of *Cucumic melo* L., which is a cucurbitane-type triterpenoid, shows an inhibitory effect on SAS cell proliferation [6].

Bitter melon or bitter gourd (*Momordica charantia* L.), belonging to the family Cucurbitaceae, is a common vegetable in the tropical areas of Asia, Africa, and South America, and has been used as a traditional medicine for a long time [7]. The medicinal value of bitter melon for its antidiabetic, anti-inflammatory, antiviral, and anticancer effects is also supported by various scientific studies [8,9,10,11]. The cucurbitane-type triterpenoids are the major bioactive constituents that are present in bitter melon and provide major contributions to its various biological activities [7]. To date, hundreds of cucurbitane-type triterpenoids have been isolated from bitter melon, and these triterpenoids contain a skeleton made up of a tetracyclic nucleus with a variety of oxygen substitutions [12]. Charantagenin D, goyaglycoside D, charantagenin E, momordicoside K and stigmasta-7,25(27)-dien-3β-ol are some of the cucurbitane-type triterpenoids isolated from bitter melon; the former two contain an –OCH_3_ substituent group in the side chain and show prominent cytotoxic effects against A549 lung cancer cells and U87 glioblastoma cells, while the latter three compounds show moderate activity [13]. Furthermore, 3β,7β-dihydroxy-25-methoxycucurbita-5,23-diene-19-al was also reported to show a significant cytotoxic effect against MCF-7 and MDA-MB-231 breast cancer cells, which are mediated through the activation of PPARγ [14], indicating the potential of *M. charantia* triterpenoids to act as anticancer agents.

Traditional solvent extraction methods are based on the solubility of materials in solvent and are mostly time-consuming, with low efficiency [15]. However, with the aid of ultrasound technology, extractions can now be finished in minutes, with high reproducibility, using lower amounts of solvents, and simplifying manipulation and workup, resulting in the higher purity of the final product. Using this method, the phytochemicals in the plant materials can be released more easily due to the effect of acoustic cavitation [16]. Similarly, microwave-assisted extraction combines the use of microwaves with traditional solvent extraction, thereby improving the migration of ions and rotation of dipoles caused by microwave energy to produce heat rapidly [17]. Tian et al. (2013) [18] found that by using the microwave-assisted technique for the isolation of triterpenoids from bitter melon, the operation was much simpler and faster than regular extraction methods, considering the absorbance value and total triterpenoid yield as a benchmark. 

Therefore, the present study aimed to analyze and quantify 10 cucurbitane-type triterpenoids by using the UHPLC–MS/MS system, to compare the effect of ultrasound- and microwave-assisted extraction methods. Moreover, the effect of these triterpenoids was also evaluated for their antiproliferative effect on SAS human oral cancer cells.

## 2. Materials and Method

### 2.1. Chemicals and Reagents

**Figure 1:**
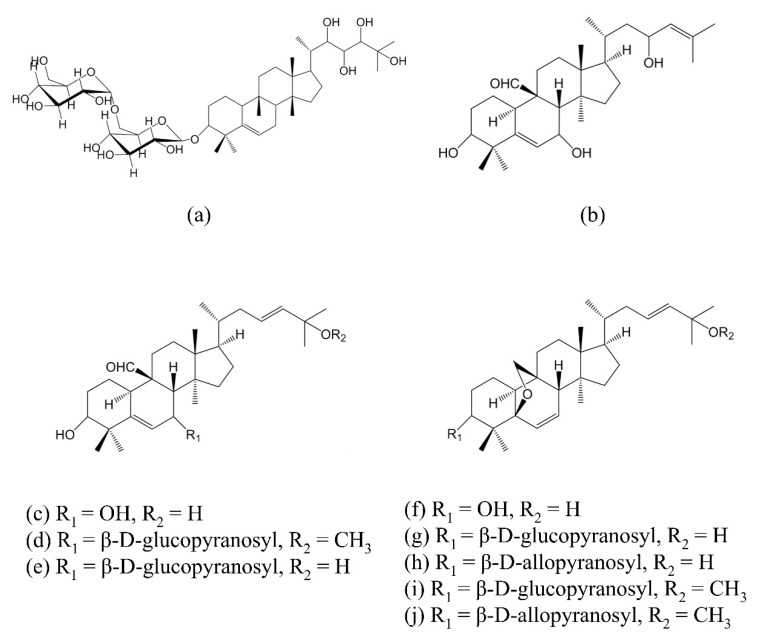
Structure of cucurbitane-type triterpenoids from *Momordica charantia*: (**a**) momordicoside A; (**b**) momordicine I; (**c**) 3β,7β,25-trihydroxycucurbita-5,23(E)-dien-19-al; (**d**) momordicoside K; (**e**) momordicoside L; (**f**) momordicoside I aglycone; (**g**) momordicoside I; (**h**) momordicoside F_2_; (**i**) momordicoside F_1_; (**j**) momordicoside G. Structures were drawn using ChemDraw 20.

Methanol (LC–MS grade and HPLC grade) was purchased from J.T.Baker (Center Valley, PA, USA), formic acid (HPLC grade) was purchased from Merck (Darmstadt, Germany), dimethyl sulfoxide (DMSO) was obtained from Sigma (St. Louis, MO, USA), and Dulbecco’s modified Eagle’s medium (DMEM) was purchased from Gibco, BRL (Grand Island, NY, USA). Fetal bovine serum (FBS), sodium pyruvate, sodium bicarbonate, and L-glutamine were purchased from Life Technologies (Grand Island, NY, USA). Ultrapure water was prepared by a Direct-Q^®^ 3 UV water purification system (Merck, Darmstadt, Germany). Triterpenoid standards (Figure 1), momordicoside L, momordicoside K, momordicine I, momordicoside F_1_, momordicoside F_2_, 3β,7β,25 trihydroxycucurbita-5,23(E)-dien-19-al, and momordicoside I were purchased from Starsci Biotech Co., Ltd. (Taipei, Taiwan). Momordicoside A was purchased from ALB Technology (Mongkok Kowloon, Hong Kong). Momordicoside G and momordicoside I aglycone were purchased from Grand Chemical (Bangkok, Thailand).

### 2.2. Sample Preparation and Extraction

#### 2.2.1. Plant Materials

The fruits of wild bitter melon (*Momordica charantia* Linn. var. *abbreviata* Ser.) Hualien No. 1 to 6 were provided by the Hualien District Agricultural Research and Extension Station, Hualien, Taiwan. The material was cleaned to remove any dirt particles with water and was lyophilized and ground to form a fine powder. The powder of bitter melon Hualien No. 4 was directly purchased from Aquavan technology Co., Ltd., Taipei, Taiwan.

#### 2.2.2. Ultrasound-Assisted Extraction

An amount of 0.5 g of bitter melon powder was weighed and mixed with 40 mL of methanol. This mixture was sonicated (Branson 5800, Brookfield, CT, USA) for 30 min at 25 °C and was then centrifuged (Beckman Allegra X-15R, Brea, CA, USA) at 4000 rpm for 15 min, according to a method established by Ma et al. (2012) [19]. After centrifugation, the supernatant was drained and collected, and the procedure was repeated five times. The collected mother liquor was concentrated until the volume was less than 40 mL, and the volume was adjusted to 40 mL with methanol.

#### 2.2.3. Microwave-Assisted Extraction

Microwave-assisted extraction was performed using a Microwave Digestion System (MARS 6, CEM, Matthews, NC, USA). An amount of 0.5 g of bitter melon powder was mixed with 50 mL of methanol in 100 mL MARSXpress plus vessels (CEM, Matthews, NC, USA). The temperature was ramped up in 5 min and was held at 80 °C for 5 min, where the oven power was set at 600 W. After this process, the samples were transferred to centrifuge tubes and centrifuged at 4000 rpm for 10 min. The final volume of the collected mother liquor was made up of methanol to 50 mL.

### 2.3. Solid Phase Extraction Process Test

The obtained extracts were then further cleaned up by reversed-phase C-18 solid-phase extraction (SPE) cartridges (500 mg/6 mL, Agela Technologies, Torrance, CA, USA). SPE cartridges were conditioned with 6 mL methanol and 6 mL ultrapure water. An amount of 0.5 mL of 100 ppb triterpenoid standards was loaded onto a preactivated SPE cartridge and then eluted out at 10% intervals for 6 mL methanol to evaluate the effect of SPE cleanup. Each fraction was collected, pooled, and concentrated. On other hand, 0.5 mL of the obtained extracts was loaded individually onto a preactivated SPE column and washed with 6 mL 30% methanol. The elution was carried out with 6 mL 100% methanol, and the fraction was collected and concentrated. The concentrates were dissolved in 1 mL LC–MS grade methanol and filtered through a 0.22 µm nylon membrane filter. Both of the concentrated fractions were injected into the UHPLC–MS/MS system to analyze the cucurbitane-type triterpenoids present in them.

### 2.4. UHPLC–MS/MS Analysis

UHPLC–MS/MS analysis was performed by using a Waters Acquity UPLC system coupled to a Xevo TQ-S tandem quadrupole MS (Waters Corporation, Milford, MA, USA). An Acquity UPLC BEH C18 column (100 × 2.1 mm, 1.7 µm, Waters Corporation, Milford, MA, USA) was used. The column temperature was maintained at 35 °C, and the injection volume was 2 μL at a flow rate of 0.3 mL/min. The mobile phase consisted of 0.01% formic acid in 5% methanol (A) and 0.01% formic acid in methanol (B). The elution was performed by using gradient programming as follows: 72% B at 0–1 min, 72–74% B at 1–2 min, 74% B at 2–6 min, 74–85% B at 6–7 min, 85% B at 7–8 min, 85–99% B at 8–9.5 min, 99% B at 9.5–10.5 min, and 99–72% B at 10.5–11 min. The instrument was operated using electrospray source ionization (ESI) in positive mode. The MS capillary voltage was set at 3.0 kV. Desolvation and cone gas flows were set at 900 and 150 L/h, and the desolvation temperature was maintained at 450 °C. Detection was performed in the multiple-reaction-monitoring mode; the transitions, cone voltage, and collision energy used are listed in Table 1. Instrument control, data acquisition, and evaluation were performed with MassLynx 4.1 software (Waters, Milford, MA, USA). The contents of 10 cucurbitane-type triterpenoids were expressed in the dried plant material (μg/g).

### 2.5. Method Validation Assay

An amount of 1 mg of each triterpenoid standard was accurately weighed and dissolved in LC–MS grade methanol to make a concentration of 1.0 mg/mL and was stored at −30 °C until required for further analysis. The serial dilution method was used to prepare different concentrations of the standards ranging from 2 μg/mL to 200 μg/mL. We further diluted the standard solutions to provide a series of concentrations until signal-to-noise (S/N) > 3 to determine the limits of detection (LOD) and S/N > 10 to determine the limits of quantification (LOQ). For evaluating intraday accuracy and precision, five samples were spiked standards with similar concentrations as that of the 10 cucurbitane-type triterpenoids in extract of Hualien No. 6 on the same day. Interday accuracy and precision were evaluated by spiking three samples over a period of three consecutive days. The final concentration levels of cucurbitane-type triterpenoids and momordicoside I aglycone were identified as 50 ppb and 10 ppb, which covered the entire specified linear range. The precision was calculated by analyzing the coefficient of variation (CV%). The accuracy was determined by analyzing percent recovery. All samples were filtered through a 0.22 µm nylon membrane filter before injecting into the UHPLC–MS/MS system.

### 2.6. Cell Culture

SAS, a high-grade tumorigenic human tongue squamous cell carcinoma, was used for preliminary experiments, which was obtained from the Japanese Collection of Research Bioresources (Tokyo, Japan) [20]. The cells were cultured in DMEM (10% FBS, 1.2 g/L NaHCO_3_, 0.5 mM C_3_H_3_NaO_3_, and 2.5 mM L-glutamine). The primary human skin fibroblasts cells (HFB cells) were kindly provided by Dr. Pan-Chyr Yang of National Taiwan University (Taipei, Taiwan). The cells were routinely maintained in DMEM supplemented with 10% FBS. All cells were grown in a 5% CO_2_ humidified incubator at 37 °C.

### 2.7. Cell Proliferation Assay

The SAS cells were seeded into a 96-well culture plate at 1.3 × 10^6^ cells/well, and HFB cells were seeded at 1.26 × 10^6^ cells/well. After incubation in DMEM at 37 °C under a humidified 5% CO_2_ for 24 h, the cells were treated with 20 and 40 μM 3β,7β,25-trihydroxycucurbita-5,23(E)-dien-19-al or momordicine I dissolved in DMSO, while the control group was treated with DMSO. Real-time monitoring of the proliferation of cells was performed with the xCELLigence system (ACEA Biosciences, Inc., San Diego, CA, USA), and the cell number was expressed as the cell index.

### 2.8. Statistical Analysis

The data are presented as the means ± standard deviation (SD). A *t*-test or one-way ANOVA with Duncan’s post hoc test was employed. A *p* value of <0.05 was considered statistically significant. Principal component analysis (PCA) was used to interpret the differences between the samples according to the cucurbitane-type triterpenoids contents of bitter melon. Statistical analysis was performed using the SPSS 21 software.

## 3. Results and Discussion

### 3.1. Solid Phase Extraction Process

Elution of 10 cucurbitane-type triterpenoids was observed with different concentrations of methanol (10–100%), as shown in Figure S1. Overall, 10 cucurbitane-type triterpenoids were not detected with elution of 10, 20, and 30% methanol. A small amount of momordicoside A was eluted in 40% methanol, while most of momordicoside A could be eluted in 50% methanol. However, momordicoside G, momordicoside F_1_, and 3β,7β,25-trihydroxycucurbita-5,23 were completely eluted in 90% methanol, while the other six cucurbitane-type triterpenoids could be eluted in 60–80% methanol. Therefore, the solid-phase extraction column was washed with 30% methanol, and 100% methanol was used as the elution buffer in subsequent sample clean-up experiments. The elution concentration of cucurbitane-type triterpenoids from bitter melon was similar to those of previous studies [21,22]. The retention time sequence was cucurbitane-type triterpenoid with five > four > three glucoside [23]. Polar compounds such as Momordica A eluted faster through reversed-phase SPE columns.

### 3.2. Method Validation

We analyzed the 10 cucurbitane-type triterpenoids from bitter melon using UHPLC–MS/MS with the multiple-reaction-monitoring (MRM) mode. A BEH C18 column with a mobile phase system, consisting of 0.01% aqueous formic acid in methanol and 100% methanol, successfully separated 10 analytes in 15 min, with a good resolution. The UHPLC–MS/MS chromatogram is shown in Supplementary Figure S2. All cucurbitane-type triterpenoids were analyzed with sodium adduct [M + Na]^+^ as the parent ion in ESI positive mode. The identification of all analytes was achieved from the retention time, molecular ion, and fragmentation pattern for the quantitative determination. The analytical method showed that the results had good linearity (*r* > 0.9917–0.9991), ranging from 2 ng/mL to 200 ng/mL, as shown in Table 2. The results reveal that the 10 cucurbitane-type triterpenoids could be reliably determined; LOD and LOQ values were 0.125–10 ng/mL and 0.25–15 ng/mL, respectively. The intraday recovery and precision of the 10 cucurbitane-type triterpenoids ranged from 91.9% to 107.8% and from 3.68% to 12.59%, respectively (data not shown). The interday precision was 7.77–14.69% (Table 2). The interday recovery of the 10 cucurbitane-type triterpenoids ranged from 85.5% to 115.3%, which indicated that the matrix effects of these analytes were negligible. All calculated intra- and interday precision rates were <15%. The results indicated good linearity and accuracy over the selected range. These LOD and LOQ results were similar to those of other LC/MS/MS detection methods in the literature [19,24], and the LOD and LOQ values were much lower than HPLC/ELSD [25]. This is the first report of the qualification of 10 terpenoids in bitter melon by UHPLC–MS/MS.

### 3.3. Comparison of Ultrasound-Assisted Extraction and Microwave-Assisted Extraction

**Figure 2:**
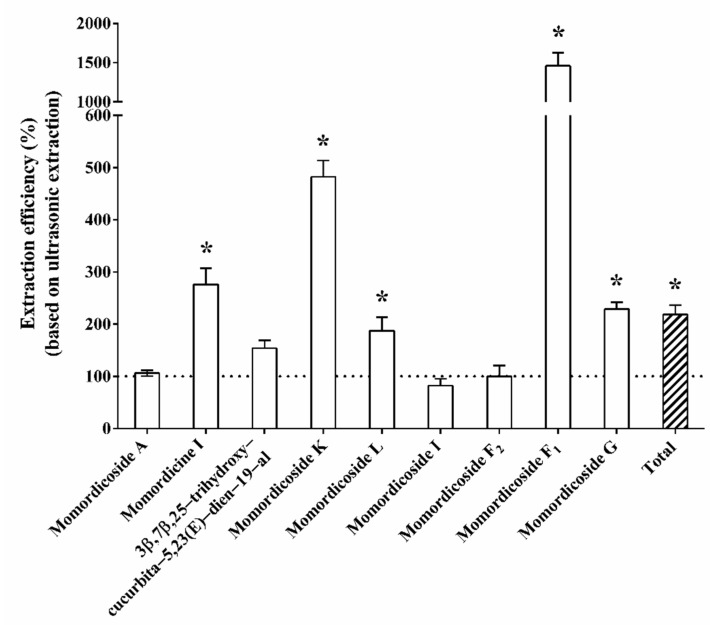
The microwave-assisted extraction efficiency of cucurbitane-type triterpenoids (based on ultrasonic extraction). The concentration of the nine triterpenoids in bitter melon extracted by microwave was divided by the concentration of the nine triterpenoids in Hualien No. 3 bitter melon extracted by ultrasound individually and presented as a percentage. All data are reported as the mean (± standard deviation) of three separate experiments. Statistical analysis was performed using a *t*-test, with significant differences determined at the level of * *p* < 0.05.

Since ultrasound-assisted extraction is simple, inexpensive, safe, and more efficient than conventional extraction techniques, the extraction of triterpenoids carried out by ultrasound was common [26]. However, when compared with microwave-assisted extraction, ultrasound-assisted extraction is less robust, and the particle size is also a critical factor [27]. Zheng et al. (2020) [28] showed that the triterpenoid yield increased as the extraction temperature changed from 40 °C to 80 °C and then decreased above 80 °C by microwave. The bitter melon extracts were heated at various temperatures, including 30, 60, and 100 °C, and the samples were collected at 5 min intervals for saponin. The levels of momordicoside F_1_, momordicoside F_2_, momordicoside I, momordicoside K, momordicoside L, 3β,7β,25-trihydroxycucurbita-5, 23(E)-dien-19-al, and momordicine I remained stable during the 5 min of treatment at 30, 60, and 100 ℃ temperatures. However, heating at 100 °C might change the levels of momordicoside F_2_, compared with those at lower temperatures. The levels of 3β,7β,25-trihydroxycucurbita-5,23(E)-dien-19-al were significantly reduced after 10 min at 100 °C [22]. Therefore, the microwave-assisted extraction condition was 80 °C in this study. Our preliminary experimental results showed that the microwave extraction time for 2, 5, and 10 min did not affect the content of total cucurbitane-type triterpenoids from bitter melon (data not shown). To compare the effects of ultrasonic extraction and microwave-assisted extraction on the amount of triterpenoid in bitter melon extract, the amounts of the content of 10 bitter melon triterpenoids were added up as total triterpenoid content, as shown in Figure 2. The triterpenoid content of bitter melon extracted by microwave-assisted extraction was significantly higher than that of bitter melon extracted by ultrasonic extraction. Among the 10 targeted triterpenoids, the contents of momordicoside L, momordicoside K, momordicine I, momordicoside F_1_, and momordicoside G were significantly higher in microwave-assisted extracted bitter melon than in ultrasonic-extracted bitter melon, while the concentrations of momordicoside A, 3β,7β,25-trihydroxycucurbita-5,23(E)-dien-19-al, momordicoside I, and momordicoside F_2_ showed no significant difference between the ultrasonic-extracted and microwave-assisted extracted group (Figure 2). This result suggested that microwave-assisted extraction had a higher efficiency in bitter melon triterpenoid extraction than ultrasound-assisted extraction. The extraction efficiency of microwave-assisted extraction is related to the mechanical effects of the rotation of molecules, which are due to a microwave-induced dipole, causing the migration of the ions, which can improve the penetration of the solvent into the matrix. This disrupts the cell wall, thereby releasing the intracellular products, permitting the dissolution of components that are to be extracted [28,29].

Microwave-assisted extraction has been used for the extraction of Ginseng and triterpene triterpenoids from the defatted residue of yellow horn (*Xanthoceras sorbifolia* Bunge) [30,31]. Chen et al. (2007) [31] found that, in comparison with shaking extraction, supercritical fluid carbon dioxide extraction, heat reflux extraction, and ultrasound-assisted extraction, microwave-assisted extraction had the highest efficiency in extracting total triterpenoid triterpenoids from *Ganoderma atrum* [30]. However, a previous study suggests that cucurbitane-type triterpenoids, such as 3β,7β,25-trihydroxycucurbita-5,23 (E)-dien-19-al and momordicine I, were extremely sensitive at 100 °C [22]; hence, temperatures below 100 °C are recommended for such compounds through the microwave-assisted extraction technique.

### 3.4. Application of Microwave-Assisted Extraction on Six Different Wild Bitter Melons

The bitter melon fruit powders of Hualien No. 1 to 6 were extracted with methanol using microwave and analyzed by the UHPLC–MS/MS system. Upon investigation, Hualien No. 4 and 5 were found to contain maximum triterpenoid concentration, at 1509.71 ± 55.97 μg/g and 1150.31 ± 52.06 μg/g, while Hualien No. 6 was observed to have the lowest content, at 90.58 ± 7.57 μg/g (Table 3). Momordicoside A was found to be the most abundant triterpenoid in Hualien No. 1, 2, 3, and 5, with concentrations of 358.59 ± 18.12 μg/g, 339.61 ± 31.92 μg/g, 195.55 ± 6.88 μg/g, and 1261.6 ± 51.54 μg/g, while momordicine I was the abundant bioactive in Hualien No. 4 (470.01 ± 25.03 μg/g) and 6 (43.93 ± 3.60 μg/g). The composition of triterpenoids was similar to that of Hualien No. 1 and 2, except the contents of momordicine I and 3β,7β,25-trihydroxycucurbita-5,23(E)-dien-19-al which were found to be higher in No. 2. In Hualien No. 3, the concentration of momordicoside L was significantly higher than that of the other five bitter melon Hualien cultivars. This is the first report on the analysis of the triterpenoid contents in different varieties of *Momordica charantia*. These data demonstrated that the compositions of triterpenoids in different bitter melon cultivars were also different.

**Figure 3:**
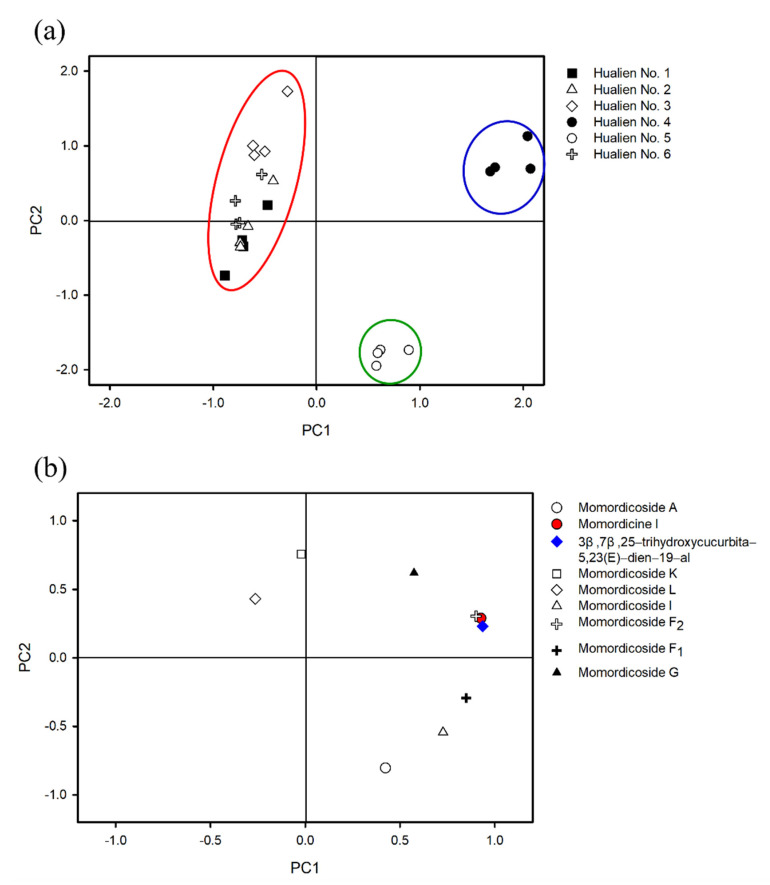
Principal component analysis (PCA) loadings and scores: (**a**) PCA score plot of PC2 versus PC1. The six kinds of bitter melon (Taiwan); (**b**) variables (nine cucurbitane-type triterpenoids) used for PCA scoring. PC1 and PC2: principal component 1 and 2.

We further analyzed the content of cucurbitane-type triterpenoids (excluding momordicoside I aglycone) of bitter melon through multivariate principal component analysis. The results show that there were three principal components with eigenvalues greater than 1. When the three principal components replaced the original variables, 93.3% of the total variation from the original data could be explained. Among the three principal components, PC1 and PC2 had the largest contribution to the total variation. Their ability to explain the total variation from the original data was 48.6% and 26.7%, respectively. Six different wild bitter melons were distributed using PC1 and PC2 principal components (Figure 3a). The results showed that the six different wild bitter melon cultivars can be divided into three groups (Hualien No. 4, No. 5, and others). The evaluation of the characteristic indicators of the PC1 and PC2 principal components indicated that the 3β,7β,25-trihydroxycucurbita-5,23(E)-dien-19-al, momordicine I, and momordicoside F_2_ variables on PC1 had greater degrees of contribution (Figure 3b). Interestingly, the contents of momordicine I (470.01 ± 25.03 μg/g), 3β,7β,25-trihydroxycucurbita-5,23(E)-dien-19-al (189.84 ± 7.80 μg/g), and momordicoside F_2_ (75.63 ± 3.33 μg/g) in Hualien No. 4 were significantly higher than those of the other five Hualien bitter melon cultivars (Table 3). Bitter melon Hualien No. 4 has been reported to have beneficial effects, such as improving metabolic syndrome in humans [32], suppressing inflammation induced by *Propionibacterium acnes* [33], and against alcoholic fatty liver by attenuating inflammatory responses and oxidative stress in mice [34]. However, the effective active compound of Hualien No. 4 has not been clarified yet, and our results seem to provide a research direction. Therefore, momordicine I and 3β,7β,25-trihydroxycucurbita-5,23(E)-dien-19-al were chosen, with relatively high content for subsequent cell experiments.

### 3.5. 3β,7β,25-Trihydroxycucurbita-5,23(E)-dien-19-al and Momordicine I Suppressed the Proliferation of SAS Cells

**Figure 4:**
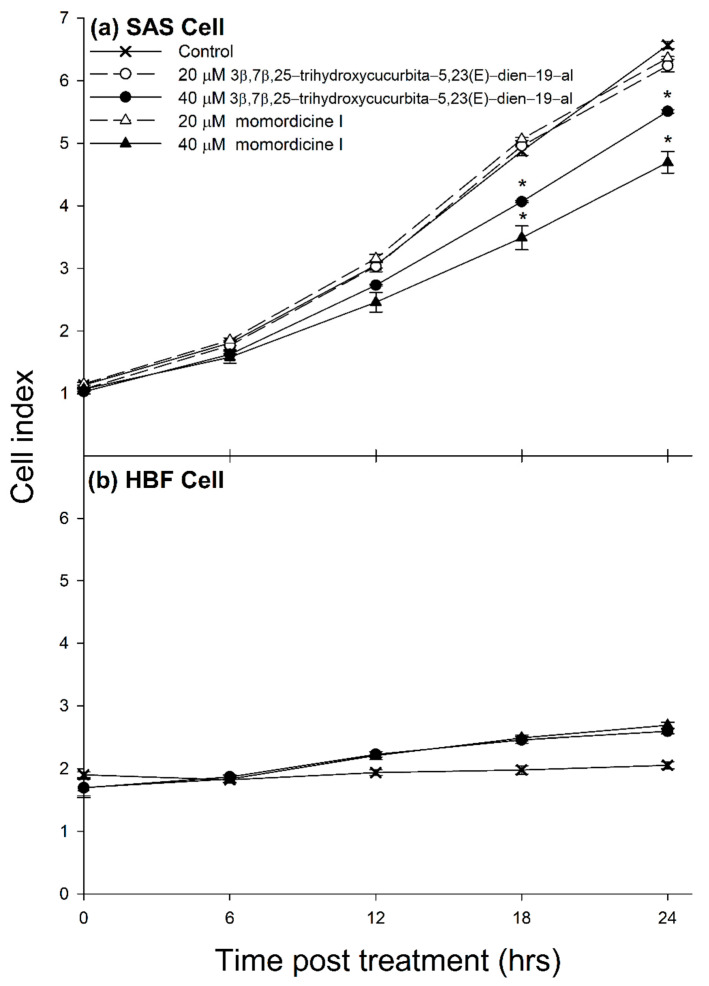
The effect of 3β,7β,25-trihydroxycucurbita-5,23(E)-dien-19-al and momordicine I on the proliferation of (**a**) SAS cell and (**b**) HFB cell. SAS cells were treated with 20 μM and 40 μM 3β,7β,25-trihydroxycucurbita-5,23(E)-dien-19-al or momordicine I for 24 h. HFB cells were treated with 40 μM 3β,7β,25-trihydroxycucurbita-5,23(E)-dien-19-al or momordicine I for 24 h. Statistical analysis was performed using a *t*-test, with significant differences determined at the level of * *p* < 0.05 versus the control group.

The effects of 3β,7β,25-trihydroxycucurbita-5,23(E)-dien-19-al and momordicine I on the cell growth of SAS cells (oral squamous cell carcinoma) were evaluated. The results indicate that 20 μM of 3β,7β,25-trihydroxycucurbita-5,23(E)-dien-19-al or momordicine I was not significantly different from the control group within 24 h (Figure 4a). Therefore, the dosage was increased to 40 μM 3β,7β,25-trihydroxycucurbita-5,23(E)-dien-19-al or momordicine I, but both were significantly lower than the cell index of the control group after 18 h. Additionally, there was no suppression of cell growth observed after the treatment of 3β,7β,25-trihydroxycucurbita-5,23(E)-dien-19-al and momordicine I in HFB cells (Figure 4b). These results suggested that 3β,7β,25-trihydroxycucurbita-5,23(E)-dien-19-al and momordicine I suppressed the proliferation of SAS cells and had no adverse effect on the cell growth of HFB cells at 40 μM. In addition, 3β,7β,25-trihydroxycucurbita-5,23(E)-dien-19-al was found to have the ability to suppress the proliferation of MCF-7 and MDA-MB-231 breast cancer [35]. Further reports also suggest that momordicine I can inhibit HNC cell (JHU022, JHU029, and Cal27) proliferation involving c-Met and downstream signaling. Studies also report that momordicine I exhibited similar activities to prevent HNC tumor growth in mice, with no apparent side effects [36]. In addition, Sur and Ray (2021) [37] found that bitter melon extract could induce cell death, inhibit cell proliferation and metabolism, and enhance the immune defense system in the prevention of OSCC in vitro and in vivo. The effect of 3β,7β,25-trihydroxycucurbita-5,23(E)-dien-19-al or momordicine I as therapeutics requires further experiments to confirm.

## 4. Conclusions

In summary, microwave-assisted extraction increases the efficiency of triterpenoid extraction in bitter melon in comparison with ultrasound-assisted extraction. Via the use of microwave-assisted extraction, bitter melon Hualien No. 4 had the highest amount of 3β,7β,25-trihydroxycucurbita-5,23(E)-dien-19-al, momordicine I, and momordicoside F_2_ among the other six bitter melon cultivars. Furthermore, 3β,7β,25-trihydroxycucurbita-5,23(E)-dien-19-al and momordicine I were found to suppress the proliferation of SAS cells but did not suppress the proliferation of HFB cells. However, further investigation is required to fully understand the effects of 3β,7β,25-trihydroxycucurbita-5,23(E)-dien-19-al and momordicine I on SAS and HFB cell lines.

## Figures and Tables

**Table 1:** The multiple-reaction-monitoring transition for 10 cucurbitane-type triterpenoids.

Compound	Molecular Weight (g/mol)	Quantitative			Qualitative		
		Parent Ions * > Product Ions (*m*/*z*)	Cone Voltage (V)	Collision Energy (eV)	Parent Ions * > Product Ions (*m/z*)	Cone Voltage (V)	Collision Energy (eV)
Momordicoside A	817.01	839 > 839	91	44	839 > 365	91	57
Momordicoside L	634.40	657 > 477	78	41	657 > 203	78	43
3β,7β,25-trihydroxycucurbita-5,23(E)-dien-19-al	472.40	495 > 495	93	13	495 > 477	93	35
Momordicoside K	648.40	671 > 203	87	43	671 > 491	87	43
Momordicine Ⅰ	472.70	495 > 495	61	3	495 > 477	61	33
Momordicoside I	618.40	641 > 337	94	46	641 > 203	94	52
Momordicoside F_2_	618.40	641 > 337	94	46	641 > 203	94	52
Momordicoside I aglycone	456.72	479 > 479	50	18	-	-	-
Momordicoside G	632.42	655 > 625	68	42	655 > 337	68	38
Momordicoside F_1_	632.42	655 > 625	17	51	655 > 337	17	57

* Positive mode [ESI^+^] [M + Na]^+^.

**Table 2:** Calibration curves, linearity assay, LOD, LOQ, accuracy, and precision of 10 cucurbitane-type triterpenoids.

Compound	Linear Range (ng/mL)	Retention Time (min)	*r*	Calibration Curve	LOD (ng/mL)	LOQ (ng/mL)	Interday Recovery (%)	Interday (CV %)
Momordicoside A	2–200	1.04	0.9989	y = 142.41 x + 148.41	0.125	0.25	100.2 ± 4.7	7.77
Momordicoside L	2–200	3.01	0.9973	y = 204.67 x − 242.72	0.50	1.00	113.1 ± 5.7	12.19
3β,7β,25-trihydroxycucurbita-5,23(E)-dien-19-al	2–200	4.26	0.9977	y = 11,142.59 x − 14,020.99	0.50	1.00	110.9 ± 6.3	14.52
Momordicoside K	2–200	6.24	0.9991	y = 213.17 x − 571.18	1.50	2.00	95.5 ± 7.3	13.09
Momordicine Ⅰ	20–200	6.72	0.9941	y = 1444.18 x + 4685.53	10.00	15.00	101.5 ± 10.6	14.21
Momordicoside I	20–200	6.88	0.9961	y = 27.70 x + 62.06	3.00	10.00	99.1 ± 6.1	12.33
Momordicoside F_2_	20–200	7.11	0.9921	y = 29.99 x − 189.06	3.00	10.00	85.5 ± 10.7	14.36
Momordicoside I aglycone	20–200	8.54	0.9917	y = 652.41 x + 479.97	3.00	10.00	103.0 ± 4.0	14.69
Momordicoside G	2–200	9.42	0.9976	y = 30.60 x + 3.14	1.50	2.00	106.5 ± 6.6	14.23
Momordicoside F_1_	2–200	9.31	0.9965	y = 107.45 x + 184.30	0.25	0.50	115.3 ± 3.7	12.82

**Table 3:** The contents of ten triterpenoids in different bitter melon cultivars.

Compound	Contents (μg/g)					
	No. 1	No. 2	No. 3	No. 4	No. 5	No. 6
Momordicoside A	358.59 ± 18.12 ^A,x^	339.61 ± 31.92 ^A,x^	195.55 ± 6.88 ^A,y^	379.37 ± 27.53 ^B,x^	1261.6 ± 51.54 ^A,w^	11.64 ± 0.60 ^B,z^
Momordicine I	24.78 ± 4.93 ^B,z^	94.08 ± 16.81 ^B,y^	141.35 ± 6.34 ^B,x^	470.01 ± 25.03 ^A,w^	155.83 ± 11.20 ^B,x^	43.93 ± 3.60 ^A,z^
3β,7β,25-trihydroxycucurbita-5,23(E)-dien-19-al	1.89 ± 0.13 ^D,z^	4.03 ± 0.14 ^C,z^	6.94 ± 0.38 ^D,E,z^	189.84 ± 7.80 ^C,x^	37.67 ± 1.36 ^C,y^	4.15 ± 0.12 ^E,z^
Momordicoside K	4.17 ± 1.31 ^C,D,y,z^	5.31 ± 1.33 ^C,y,z^	9.71 ± 2.05 ^D,x^	5.71 ± 0.47 ^E,y^	3.40 ± 0.52 ^D,z^	3.95 ± 0.65 ^E,y,z^
Momordicoside L	10.15 ± 2.03 ^C,x,y^	12.32 ± 3.15 ^C,x^	61.15 ± 5.32 ^C,w^	7.16 ± 1.12 ^E,z^	14.54 ± 2.26 ^D,x^	10.74 ± 1.90 ^B,x,y^
Momordicoside I aglycone	N. D. *	N. D.	N. D.	N. D.	N. D.	N. D.
Momordicoside I	1.15 ± 0.18 ^D,z^	0.60 ± 0.71 ^C,z^	3.56 ± 0.62 ^E,F,y^	9.84 ± 0.73 ^E,x^	17.66 ± 0.85 ^D,w^	1.47 ± 0.38 ^F,z^
Momordicoside F_2_	3.06 ± 0.42 ^D,z^	3.30 ± 0.30 ^C,z^	6.14 ± 0.28 ^D,E,y^	75.63 ± 3.33 ^D,w^	11.44 ± 0.81 ^D,x^	6.27 ± 0.32 ^D,y^
Momordicoside F_1_	0.43 ± 0.10 ^D,y,z^	0.42 ± 0.15 ^C,y,z^	0.65 ± 0.18 ^F,y^	1.27 ± 0.24 ^E,x^	1.44 ± 0.23 ^D,x^	0.24 ± 0.16 ^F,z^
Momordicoside G	6.68 ± 2.57 ^C,D,z^	6.36 ± 2.68 ^C,z^	7.74 ± 1.51 ^D,z^	11.49 ± 1.74 ^E,y^	6.11 ± 0.63 ^D,z^	8.20 ± 2.11 ^C,z^
Total	410.91 ± 21.08 ^y^	466.04 ± 48.91 ^y^	432.79 ± 16.52 ^y^	1150.31 ± 52.06 ^x^	1509.71 ± 55.97 ^w^	90.58 ± 7.57 ^z^

* Not detected. Results expressed as mean ± standard deviation (*n* = 4). ^A–F^ The different capital letters in the same column indicate a significant difference between compounds (*p* < 0.05). ^w–z^ The different lower case letters in the same row indicate a significant difference between cultivars (*p* < 0.05).

## Data Availability

Data is contained within the article or Supplementary Materials.

## Supplementary Materials

The following are available online at https://www.mdpi.com/article/10.3390/foods11050729/s1, Figure S1: Solid-phase extraction process of (a) Momordicoside A, (b) Momordicoside L; (c) 3β,7β,25-trihydroxycucurbita-5,23(E)-dien-19-al, (d) Momordicoside K, (e) Momordicine I, (f) Momordicoside I aglycone, (g) Momordicoside G, (h) Momordicoside F1, (i) Momordicoside I, and (j) Momordicoside F2, Figure S2: LC–MS/MS chromatogram of 10 cucurbitane-type triterpenoids 100 ppb mix standard.
