# Supplementary material for: Microwave- and Ultrasound-Assisted Extraction of Cucurbitane-Type Triterpenoids from Momordica charantia L. Cultivars and Their Antiproliferative Effect on SAS Human Oral Cancer Cells
Source: Foods. 2022 Mar 1;11(5):729. doi: 10.3390/foods11050729 (PMC8909074; doi:10.3390/foods11050729)
Supplement: Supplementary file 1 [file foods-11-00729-s001.zip › foods-1570871-supplementary.pdf]

Supplementary material

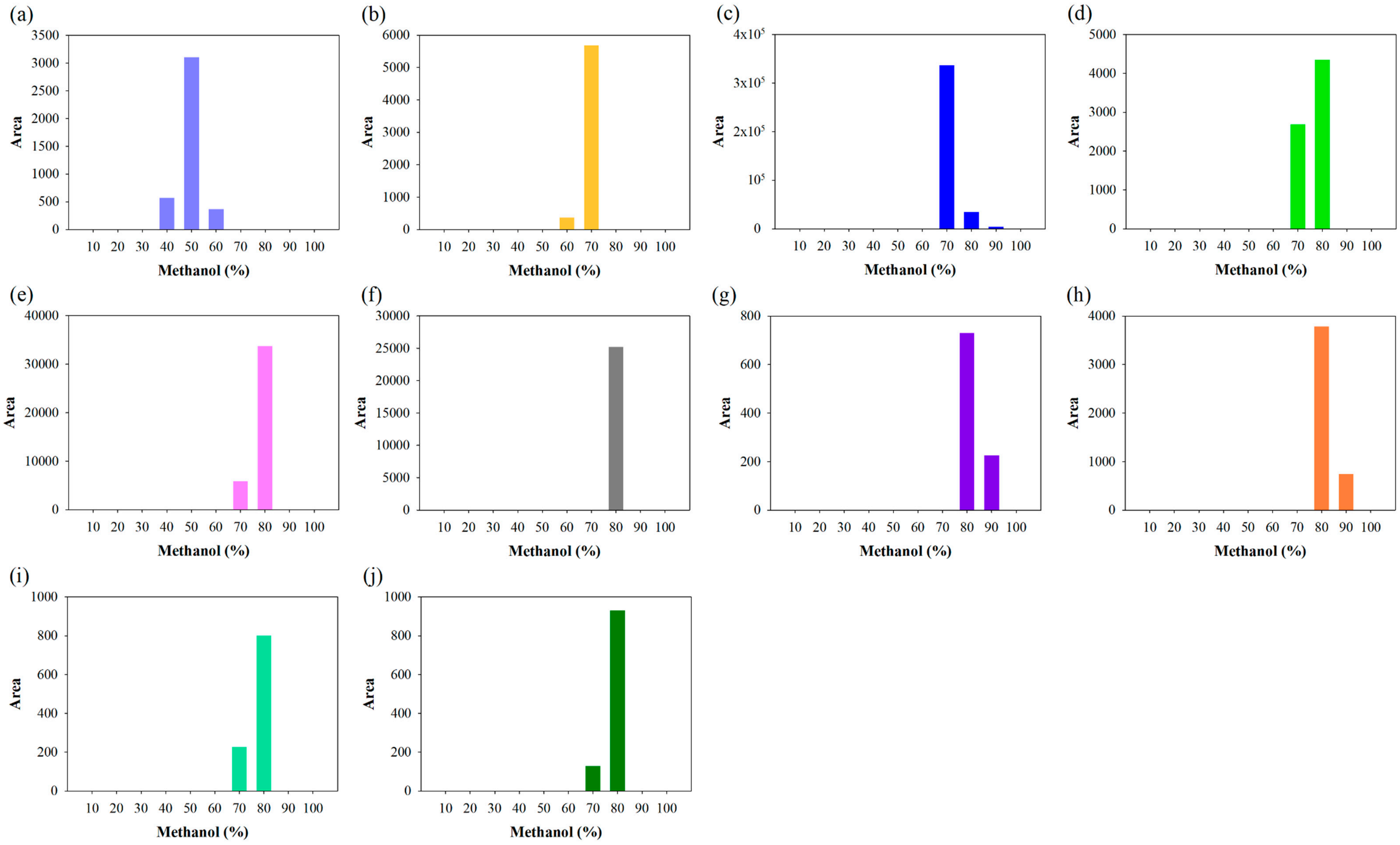

Figure S1. Solid phase extraction process of (a) Momordicoside A; (b) Momordicoside L; (c) 3β,7β,25-trihydroxycucurbita-5,23(E)-dien-19-al; (d) Momordicoside K; (e) Momordicine I; (f) Momordicoside I aglycone; (g) Momordicoside G; (h) Momordicoside F_1; (i) Momordicoside I; (j) Momordicoside F_2.

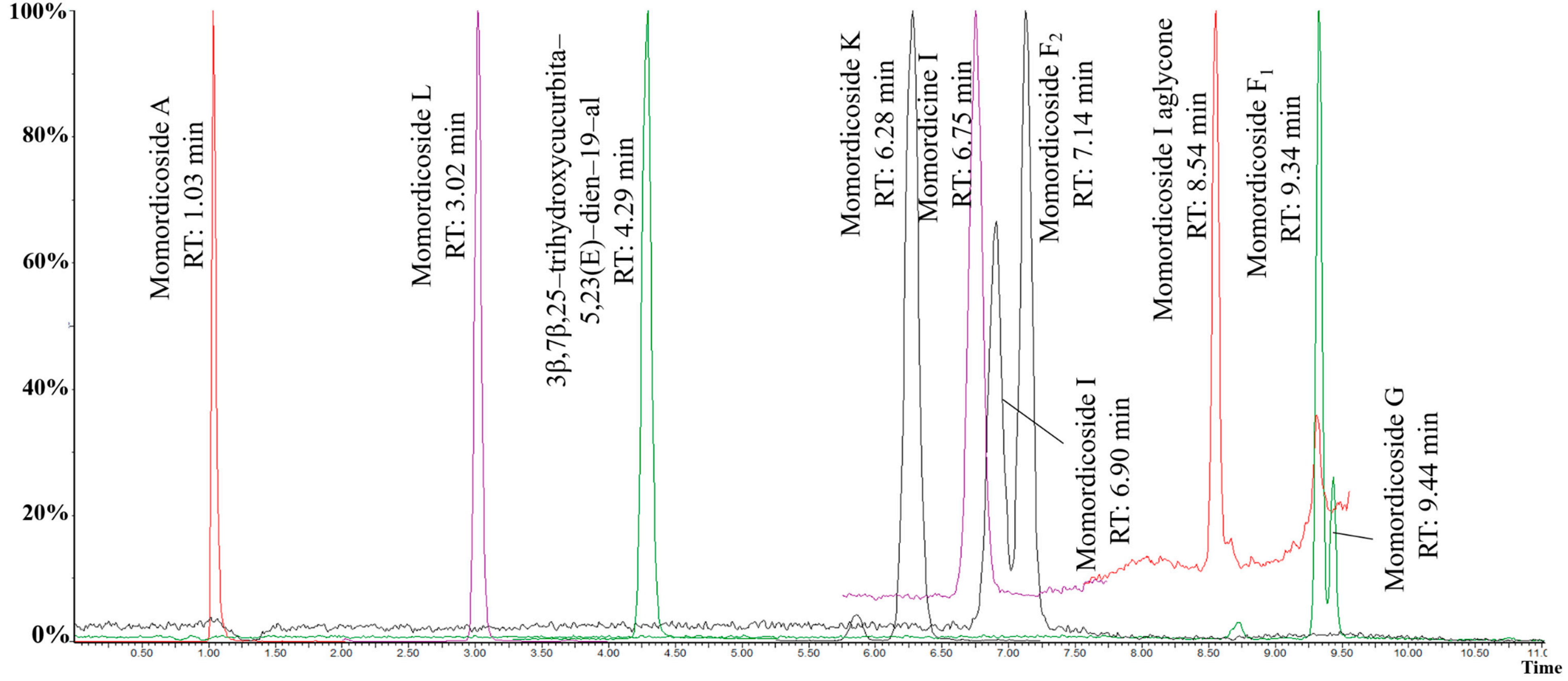

Figure S2. LC–MS/MS chromatogram of ten cucurbitane-type triterpenoids 100 ppb mix standard.
